# Case Report: Two years of compassionate use with Olipudase-alfa in a child with neurovisceral acid sphingomyelinase deficiency

**DOI:** 10.3389/fped.2024.1518344

**Published:** 2025-01-06

**Authors:** Federica Deodato, Sara Boenzi, Benedetta Greco, Alessia Graziosi, Carlo Dionisi-Vici

**Affiliations:** Division of Metabolic Diseases and Hepatology, Ospedale Pediatrico Bambino Gesù, IRCCS, Rome, Italy

**Keywords:** acid sphingomyelinase deficiency, ASMD, acute neurovisceral phenotype, chronic neurovisceral phenotype, Niemann-Pick disease type A, Olipudase-alfa, Lyso-sphingomyelin, Lyso-SM

## Abstract

Acid sphingomyelinase deficiency (ASMD) is a rare, progressive lysosomal storage disorder resulting from a deficiency in acid sphingomyelinase, leading to sphingomyelin accumulation and multi-organ damage. ASMD presents a broad phenotypic spectrum with a continuum of severity, making it challenging to predict the phenotype in very young children and differentiate between acute and chronic neurovisceral disease. No disease-specific treatments existed for ASMD. Recently, Olipudase-alfa, an intravenous enzyme replacement therapy, has been approved for non-neurological manifestations based on clinical trial results showing significant improvements. This report details the compassionate use of Olipudase-alfa in a 8-month-old boy. At baseline, he exhibited hepatosplenomegaly, elevated transaminases, and normal developmental milestones, consistent with a chronic neurovisceral phenotype. The treatment commenced at 8 months of age, escalating from 0.03 mg/kg to 3 mg/kg bi-weekly. Throughout the two-year period, the child tolerated the therapy well, with no severe adverse events reported. Notable clinical outcomes included a significant reduction in spleen and liver size, normalization of liver function tests, and stabilization of the lipid profile. The biomarker Lyso-sphingomyelin significantly reduced but never normalized, while oxysterols completely normalized. In the following months, the patient exhibited neurocognitive regression, allowing to define an acute neurovisceral phenotype. Although not impacting on the neurological manifestations, treatment with Olipudase-alfa strikingly improved the child's visceral symptoms, contrasting with the typical progressive decline seen in untreated patients. This report highlights the importance of early intervention, even in patients with neurovisceral phenotypes, as it can enhance quality of life for both patients and their families. Our findings advocate for reconsidering treatment eligibility criteria based solely on clinical phenotype definitions, highlighting the need for a tailored approach in ASMD management.

## 1 Introduction

Acid sphingomyelinase deficiency (ASMD) is a rare multisystemic, progressive lysosomal storage disease with an age of onset varying from birth to adulthood. ASMD is due to the deficiency of acid sphingomyelinase (ASM; E.C.3,1.4.12), required to degrade sphingomyelin, into ceramide and phosphocholine (1). Insufficient ASM activity results in the abnormal accumulation of sphingomyelin in the cells of the monocyte-macrophage system. Over time, sphingomyelin buildup causes progressive cell damage and impairment of multiple organs, i.e., spleen, liver, lungs, lymph nodes, bone marrow, and variably the central nervous system.

ASMD is caused by mutations in *SMPD1 gene*, and is inherited as an autosomal recessive trait. To date, over 250 pathogenic variants of *SMPD1* have been described, including missense, nonsense, frameshift, insertions, deletions, and intronic variants [see The *Human Gene Mutation Database; (*http://www.hgmd.org*)]*. The type of *SMPD1* mutation reflects the level of residual ASM activity and influences the severity of clinical manifestations. However, due to the high frequency of rare and private mutations, genotype/phenotype correlation may be difficult (2).

The clinical phenotype of ASMD is highly variable in terms of age of onset and severity of manifestations. Historically, patients with ASMD have been categorized as Niemann-Pick disease (NPD) A and B based on disease severity and the presence or absence of neurologic symptoms. Patients designated as NPD A, now classified as the infantile acute neurovisceral type, are the most severely affected, showing hepatosplenomegaly in infancy and rapidly progressive neurodegeneration within the first year of life, with death typically occurring by 3 years of age. In contrast, NPD B, now classified as the chronic visceral type, has a variable disease course, with a broad spectrum of visceral signs without neurological manifestations. The term I “intermediate form” or “variant NPD AB”, now classified as the chronic neurovisceral type, defines the phenotype showing a combination of non-neurological and mild-to-severe neurological manifestations (3).

Until early 2022, no disease-specific treatments for ASMD were available. Based on the results of clinical trials in adult and pediatric patients revealing clinically meaningful improvements in multiple domains (4, 5), Olipudase-alfa, an intravenous enzyme replacement therapy (ERT) not crossing the blood-brain barrier, was recently approved in several countries to treat the non-neurologic manifestations of ASMD.

Here, we report on two years of treatment of a 6-month-old child enrolled in the compassionate use program for Olipudase-alfa for patients with chronic ASMD (NCT04877132).

## 2 Case description

The propositus is the first child of non-consanguineous, healthy Caucasian parents. He was born at 40 weeks after an uneventful pregnancy by cesarean section due to fetal tachycardia. Birth weight was 3,820 g, length 51 cm, and head circumference 35 cm. The Apgar score was 9–10. Due to fetal tachycardia, he was admitted to the neonatal intensive care unit. ECG, Holter ECG, and echocardiography were normal. At birth, newborn hearing screening was abnormal and a macular cherry-red spot was detected on eye examination. The auditory brainstem evoked responses (ABRs) revealed mild hypoacusis. To further investigate these abnormal findings, he was referred to a tertiary hospital at 4 months of age. The physical examination showed the liver 2 cm below the right costal margin, and the spleen 1 cm below the left costal margin. He was able to smile and follow an object with his eyes, and he could hold up his head, and grab and hold toys. Weight, length, and head circumference were at the 50th, 15th, and 50th percentiles, respectively. Fundoscopy confirmed the cherry red macula. Abdominal ultrasound showed hepatomegaly (10 cm in the mid-clavicular line) with normal echogenicity, and splenomegaly (8.3 cm). Laboratory workup showed increased transaminases (AST/ALT 454/347 UI/L respectively), while blood count, albumin, prothrombin time, INR, bilirubin, and lipid profile were normal. Based on clinical suspicion of a lysosomal disease, an enzyme study was carried out. The activity of β-hexosaminidase, β-galactosidase, and α-neuraminidase in leucocytes was normal, while ASM activity in dried blood spot was 0.03 µmol/L/h (normal values >1.3). *SMPD1* gene analysis detected a homozygous pathogenic variantNM_000543.5:c.739G>A, p. Gly247Ser. A brain MRI showed mild and nonspecific enlargement of subarachnoid spaces in the frontotemporal regions. Electroencephalogram (EEG) and Visual evoked potentials (VEPs) were normal.

He was first evaluated in the Metabolic Unit at Bambino Gesù Children's Hospital when he was 6-months old. Weight, length, and head circumference were at the 25–50th, 10–25th, and 95th percentiles, respectively. He was alert, smiling, and capable of eating and swallowing normally. He could follow moving objects with his eyes, hold up his head, and sit for a few seconds, showing the ability to grab, and hold toys bringing them to his mouth. In a prone position, the child raised his head and tried to grasp an object placed in front of him. Griffiths Scales highlighted normal developmental functioning, with a developmental quotient (DQ) of 108 and an age equivalent (AE) of 6.5 months. The specific developmental subdomains showed scores in line with age expectations: locomotor DQ = 92, AE = 5.5; personal-social DQ = 116, AE = 7, hearing and speech DQ = 108, AE = 6.5, eye and hand coordination DQ = 125, AE = 7.5, performance DQ = 100, AE = 6. EEG showed normal electrical activity.

Based on the overall clinical condition he was classified as having a chronic neurovisceral phenotype. Therefore, we proposed access to the compassionate use program for Olipudase-alfa for patients with chronic ASMD. The aim of the program was to provide access to therapy before registration and availability of the commercial product in the patient's country.

The treatment was approved by the Local Ethics Committee (approval number 1592), and informed consent was obtained from the parents.

### 2.1 Treatment

#### 2.1.1 Dose escalation and maintenance phase

In December 2021, when the child was eight months old, bi-weekly recombinant enzyme administration was started. According to the investigator's brochure, Olipudase-alfa was initiated at 0.03 mg/kg and titrated to 3 mg/kg over 16 weeks. The initial infusion rate was 0.1 mg/kg/h, and gradually increased with steps every 20 min, from 0.1 mg/kg/h, 0.3 mg/kg/h, 0.6 mg/kg/h, until 1 mg/kg/h until completion of the infusion volume.

Since then, the standard dose of 3 mg/kg every two weeks has been consistently maintained. A single dose was missed, between the 29th and 30th infusion, because the child was hospitalized for RSV bronchiolitis.

#### 2.1.2 Safety and adverse events

No hypersensitivity reactions occurred during the dose escalation or in the following months of treatment, with no premedication. At week 4, the day after the third infusion (dose 0.3 mg/kg) a brief episode of increased body temperature (T max 38°C), resolved with paracetamol administration, and two episodes of vomiting occurred. No increase of AST/ALT or other biochemical abnormalities occurred, and the dose escalation continued without any other adverse events. After three months of treatment, low titer IgG antidrug antibodies developed (titer 1:50), becoming negative three months later.

### 2.2 Results

#### 2.2.1 Spleen/liver

**Figure 1:**
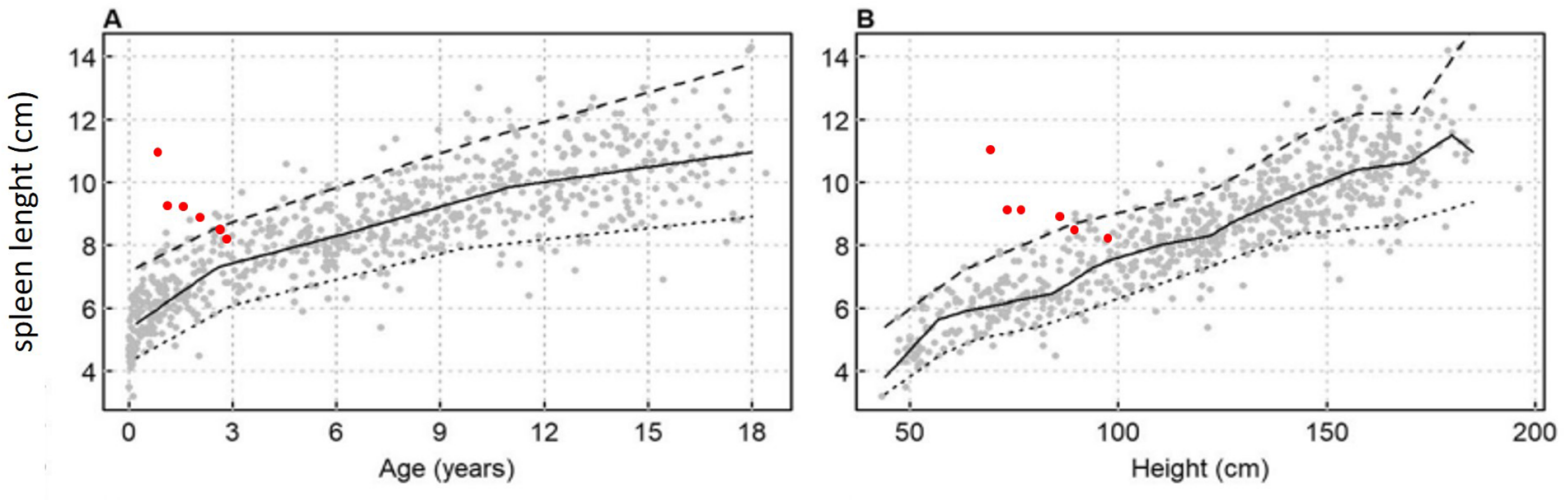
Spleen length against **(A)** age and body height **(B)** according charts for pediatric European Caucasian population spleen length is expressed in cm, with smoothing lines for the 5th, 50th and 95th percentiles (6).

The liver and spleen were assessed with abdominal ultrasound, avoiding the need for sedation for MRI. Spleen size was measured by calculating the maximum diameter between the superomedial and inferolateral points, and the percentile of length was evaluated for age and body height according to normative sonographic dimensions in the Central European pediatric population (6). At baseline the spleen diameter was 11 cm (>95th percentile for age and height). Spleen diameter was 9.5 and 9 cmafter 3 and 12 months of treatment, respectively. In the following months, spleen size consistently decreased, eventually reaching normal size (8 cm) at the last evaluation (50–95th percentile for age and height) (Figure 1). Sonographic evaluation of the liver at baseline showed hepatomegaly with a smooth liver surface and increased parenchymal echogenicity. A minute subcentimetric sparing area of the fourth segment with preserved vascular scaffolding was evident, no longer visible after three months of therapy. After six months hepatomegaly and hyperechogenecity were reduced. At the last evaluation, the liver showed normal size, with only minimal hyperechogenicity.

#### 2.2.2 Laboratory assessments

**Figure 2:**
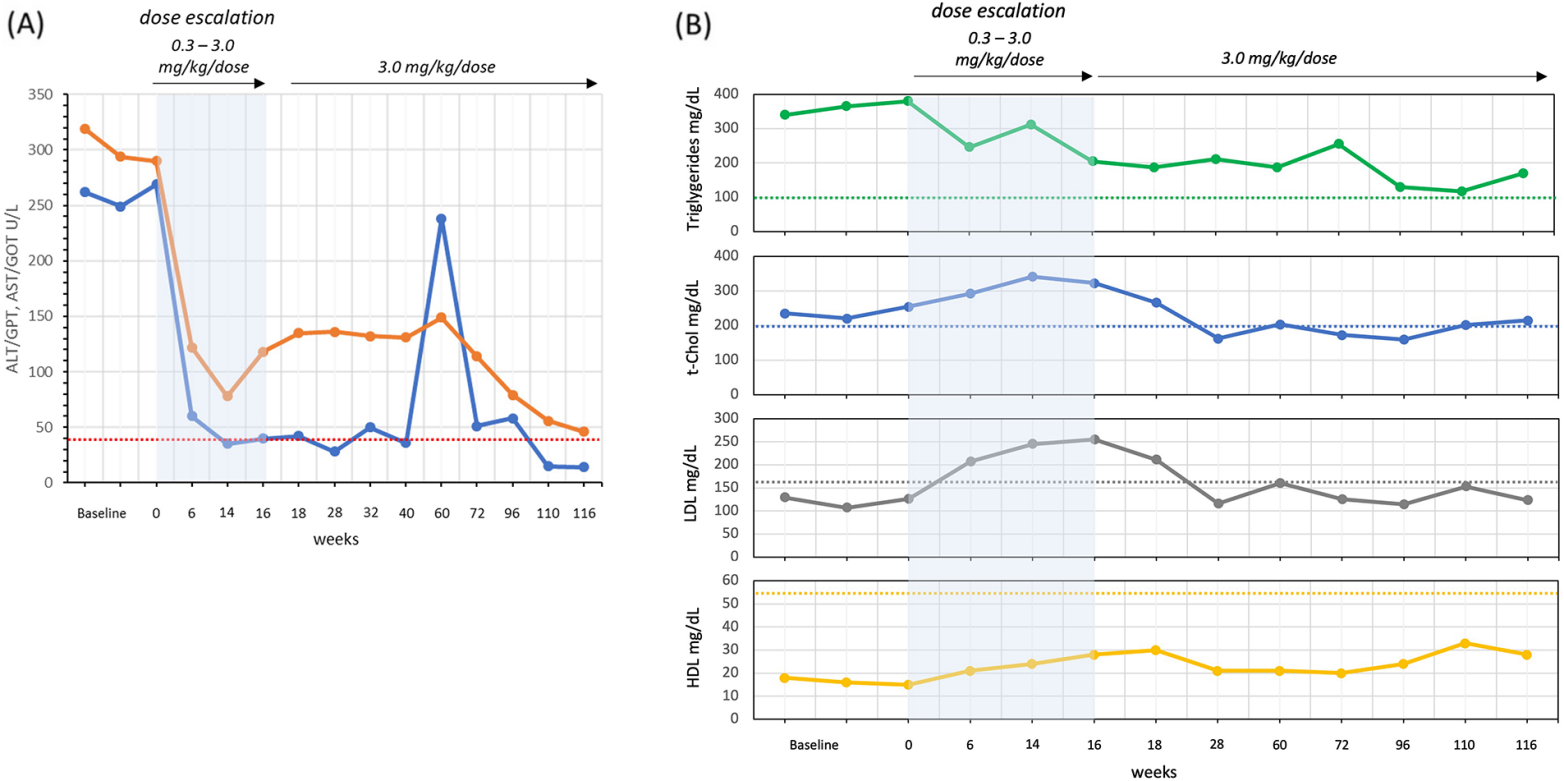
Liver function: ALT/GPT (blue line) and AST/GOT (orange line) **(A)** and lipid asset: triglycerides, total cholesterol (t-Chol), LDL and HDL **(B)** reference limits in dashed lines: ALT/GPT <41 U/L, AST/GOT <40 U/L, t-Chol <200 mg/dl, LDL <160 mg/dl, and HDL >55 mg/dl.

Liver function was evaluated during the 2 years of therapy. ALT/GPT and AST/GOT were 6–7 times above the upper limits at baseline. The concentration of ALT/GPT decreased to normal values, while AST/GOT remained slightly above the upper limit (Figure 2A). Bilirubin was always normal.

The lipid profile was evaluated by analyzing total cholesterol (t-Chol), low density lipoprotein (LDL), high density lipoprotein (HDL) and triglycerides (Figure 2B). At baseline, LDL concentration was below the upper limit of 200 mg/dl and, after an increase at the end of the dose escalation, settled within the control range 12 weeks after the end of dose escalation. t-Chol, HDL and Triglycerides were abnormal before starting therapy, and all three remained slightly outside the specific control range after two years.

**Figure 3:**
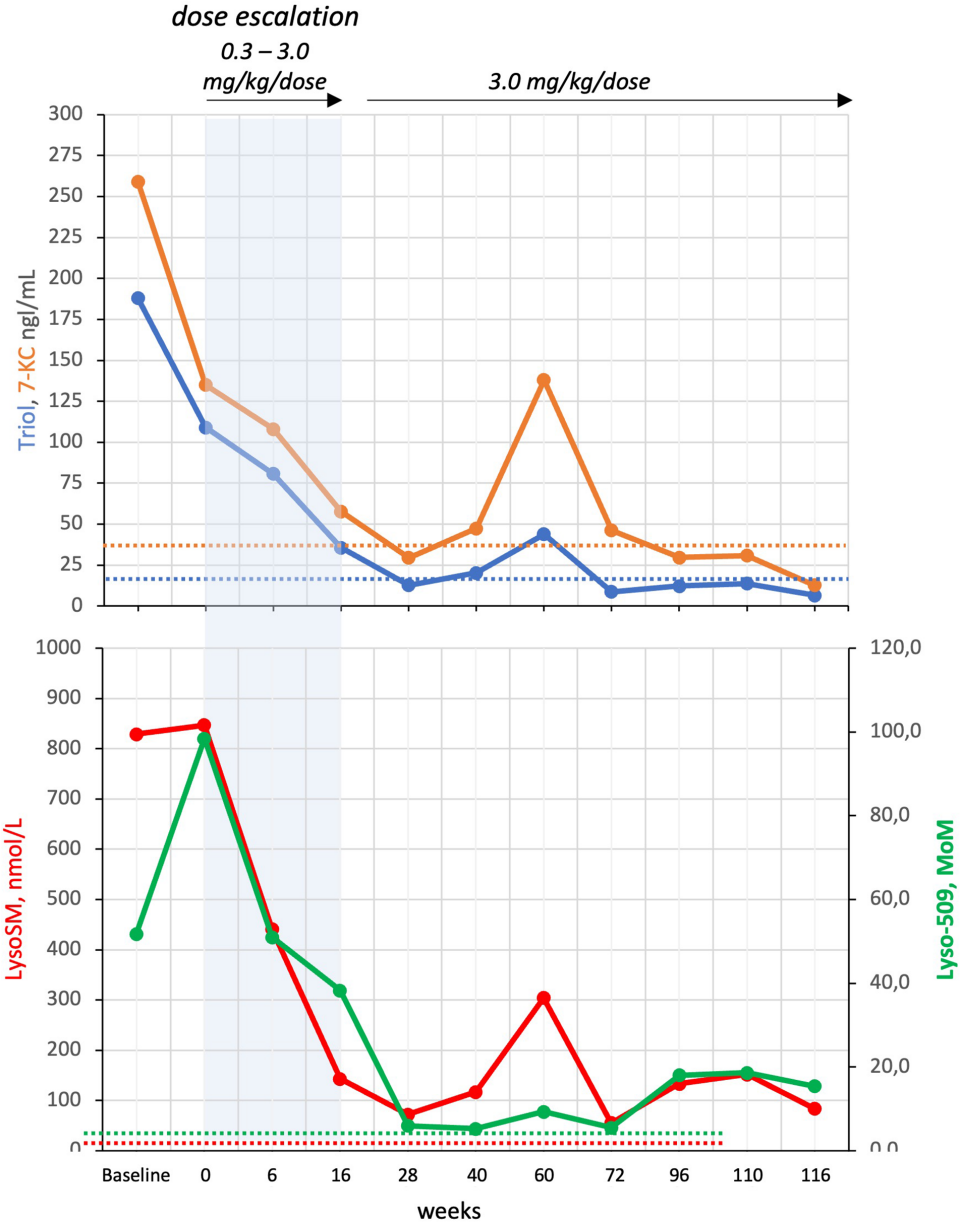
Biomarkers concentration over time during therapy. Reference upper limits in dashed lines: Triol 23.6 ng/ml, 7-KC 40.1 ng/ml, Lyso-SM 15.0 µmol/L, and Lyso-509 4.6 MoM.

The specific ASMD biomarker Lyso-sphingomyelin (Lyso-SM) together with the non-specific biomarkers N-palmitoyl-O-phosphocholineserine, also called Lyso-sphingomyelin-509 (Lyso-509), and the two oxysterols, 7-ketocholesterol (7-KC) and cholestan-3β,5α,6β-triol (Triol), were evaluated in plasma before starting therapy and at several points over the two years (Figure 3) (7). The levels of Lyso-SM and Lyso-509, which were significantly high at baseline (15 and 12 times respectively), immediately reduced after the first dose of 0.03 mg/kg, continuing to decrease but never reaching normal values. After two years, the concentrations of Lyso-SM and Lyso-509 were 5 and 3 times the upper limits respectively.

Plasma oxysterols, which were 3–4 times higher at baseline, reached normal values after the two years of therapy.

#### 2.2.3 Growth

**Figure 4:**
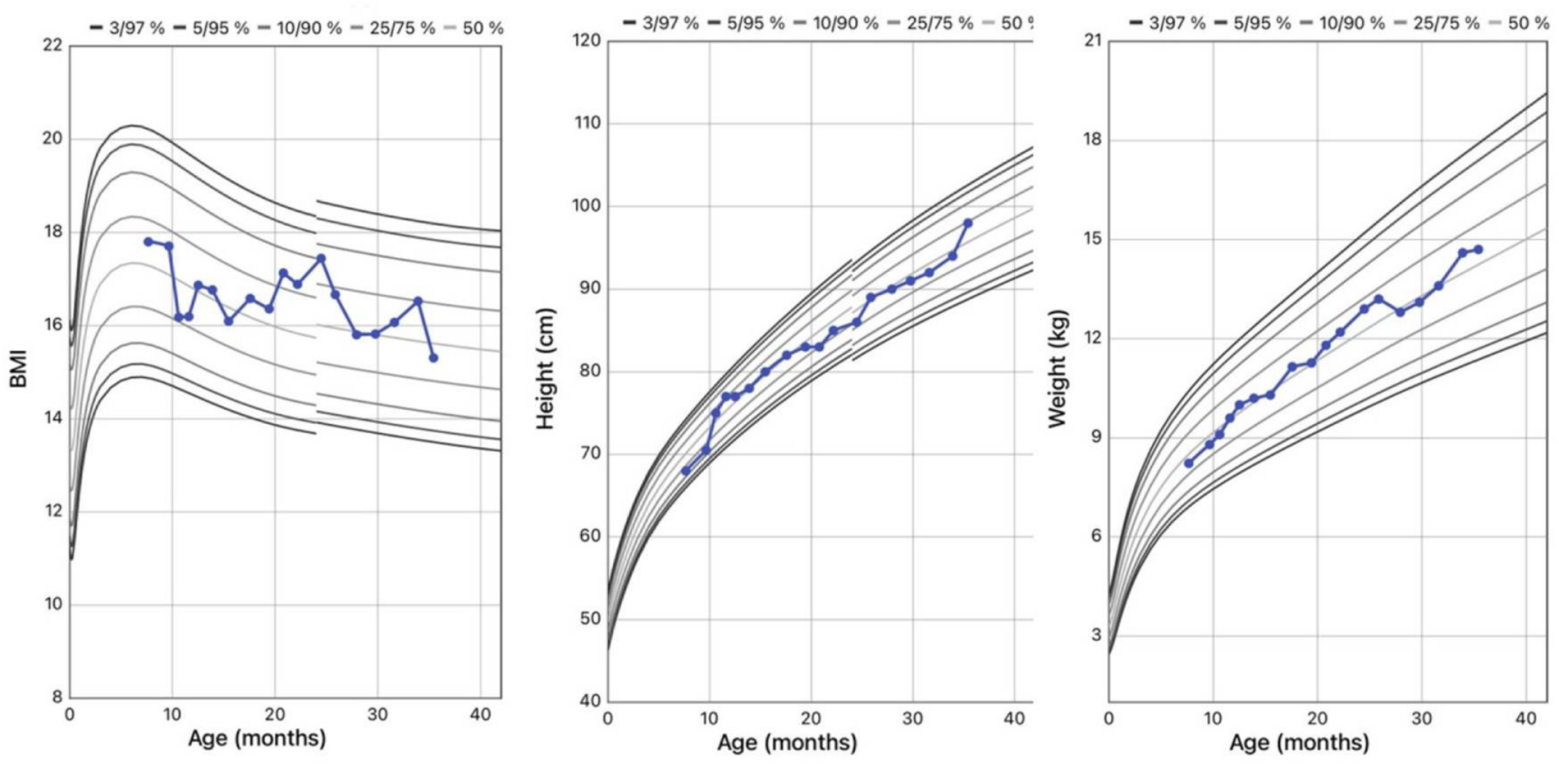
WHO growth charts for body mass index (BMI), height, and weight of the patient.

Body weight and BMI were around the 50th percentile at diagnosis, and remained stable through the years. His height was at the 25th percentile at onset, reaching the 75th at the last evaluation (Figure 4).

#### 2.2.4 Neurological outcome ant timeline of regression

During the first year of life, the patient continued to achieve developmental milestones, although he presented a delay in gross motor skills. He demonstrated good communicative intent and environmental engagement, smiling and babbling, maintaining appropriate eye contact and responding to his name. Showing interest in the provided play materials, the child grasped and transferred objects between hands, intentionally producing sounds. He achieved the ability to sit for a few minutes at the age of 12 months. No swallow or sleep disturbances were present. At 15 months, after a COVID infection, he progressively lost acquired motor skills. Psychomotor regression began at 17 months, with gradual loss of sitting ability, grasp, and babbling. While maintaining eye contact and social smiling, his overall engagement decreased, and he made no attempt to grasp objects even when within reach. A brain MRI at 18 months showed hypomyelination with T2-hypointensity and T1-hyperintensity of both thalami. By the age of 19 months, he developed strabismus, but was still capable of controlling his head, maintaining good eye contact, and swallowing normally. By the age of 22 months, he lost head control, reduced spontaneous movements and lost interest in his surroundings. Two months later the child developed seizures, which were treated with levetiracetam, and a preemptive gastrostomy was performed, before he completely lost the ability to swallow. By the age of 30 months, he was almost unresponsive and was exclusively fed through gastrostomy.

## 3 Discussion

ASMD is a severe, life-threatening disease with no specific treatments available until 2022. Clinical trials demonstrated that enzyme replacement treatment with Olipudase-alfa led to clinically significant improvements in multiple endpoints for both adult (4) and in pediatric patients with chronic ASMD (5). Recently, Olipudase-alfa has been approved in several countries for treating the non-neurologic manifestations of ASMD. However, limited data exists outside clinical trials (8, 9) and no information is available regarding very young children with a severe neurovisceral phenotype. Here, we report the results of two years of compassionate treatment of an 8-month-old ASMD child.

ASMD presents a broad phenotypic spectrum with a continuum of severity, making it challenging to predict the phenotype in very young children and differentiate between acute and chronic neurovisceral disease. In our patient, ASMD was diagnosed at four months of age, with a cherry red spot, observed since birth, a characteristic ophthalmological finding in several neuronal lipid storage disorders- including Tay–Sachs disease, Sandhoff's disease, GM2 gangliosidosis, galactosialidosis, and neuraminidase deficiency—present in all NPD A, and in one-third of individuals with NPD B (10, 11). Enzyme activity and molecular analysis confirmed the diagnosis. The child was referred to our center at six months of age, in concomitance with the initiation of the compassionate use program for Olipudase-alfa. The exclusion criteria for pediatric patients aged <3 years included a clinical diagnosis of infantile-onset ASMD (NPD A) with evidence of developmental delay, or homozygosity for specific *SMPD1* gene mutations (R496l, L302P, P330fs*52 or any combination of these mutations). In our patient, genetic testing revealed the presence of the homozygous variant, c.739G>A (p.G247S), also known as G245S due to a difference in cDNA numbering. This missense variant, previously described in a few patients, was reported to be associated with the chronic phenotype (NPD B) when present in compound heterozygosity (12, 13) and, in a single homozygous Italian child, with an acute neurovisceral phenotype (NPD A) (14).

Given the young age, the absence of psychomotor delay, and the genotypic distinction from the exclusion criteria, our child was enrolled in the compassionate program, after ethical committee approval and after informing parents of the uncertain phenotype.

No adverse events occurred in the dose escalation phase or during the two years of treatment.

Clinically, we observed significant reductions in spleen and liver size, alongside improvements in liver steatosis. After two years of therapy, transaminases, lipid profile, and disease-related biomarkers showed a significant improvement. ALT/GPT normalized, while AST/GOT levels decreased but remained above the upper limit of the control range. Noteworthy ALT/GPT temporarily increased at week 60, coinciding with a missed dose during hospitalization for bronchiolitis. The lipid profile showed a transient worsening during dose escalation, then improving to borderline/normal when the therapeutic regimen reached 3.0 mg/kg/dose.

Oxysterols normalized after two years of therapy, with Triol decreasing more slowly compared to 7-KC, probably meaning a decrease in cellular oxidative stress (15). Unlike oxysterols, the two more specific biomarker, Lyso-SM and Lyso-509, showed a less pronounced decrease during therapy, never reaching reference normal concentrations. Notably, the missed dose of Olipudase-alfa at week 60 corresponded with a transient elevation in Lyso-SM and both oxysterols. Alongside the normalization of organomegaly, liver function, lipid profile, and marked reduction of disease-related biomarkers, our patient achieved normal growth, did not experience recurrent lung infections, bleeding, or the need for transfusions or multiple hospitalizations.

After the age of 15 months, the patient stated to exhibit a neurocognitive regression, consistent with an acute neurovisceral phenotype (NPD A). Therefore, this case provides further evidence of the p.G247S variant being correlated with acute phenotype in homozygosity. Children with this phenotype typically display early and rapidly progressive hepatosplenomegaly, liver dysfunction, ascites, and/or cholestasis (16–18). Transaminase levels are always elevated from early months, with bilirubin elevated in most patients. Frequently they have low platelets with high risk of bleeding. Feeding problems usually arise at a median age of 9 months, with failure to thrive at a median age of 10 months until cachexia. Respiratory symptoms typically manifest by 9 months, and neurologic deficits become around the same time, with most patients succumbing by three years of age.

Despite the known inability of ERTs to cross the blood-brain barrier, they are widely used in chronic neuronopathic lysosomal diseases, addressing peripheral organs and reversing the natural history of the disease. In acute Gaucher disease (GD2), usually characterized by a very early significant neurological impairment, the use of ERT is commonly not indicated (19). However, the distinction between GD2 and GD3, may be not always clear (20) and the use of ERT has been debated and it has been considered in selected cases, at least until the phenotype is clarified, discussing with families the potential benefits and limitations (21).

Although ERT does not halt neurodegeneration, our patient showed a markedly positive impact on visceral disease manifestations, as seen in children with chronic ASMD, indicating an overall benefit on the natural disease course. Unlike untreated acute and chronic neurovisceral individuals (18), our patient normalized hepatosplenomegaly, liver function, without signs of progressive interstitial pulmonary disease. This improvement in non-neurological disease characteristics, clearly alleviated the disease burden/course, and positively impacted clinical- and psychological well-being. Caregiver's emotional exhaustion often stems from the constant anticipation of ASMD progression while managing daily symptoms such as recurrent vomiting or persistent nausea (22). Few studies have assessed the impact of ASMD on health-related quality of life (QoL) and its effects on patients and families. NPD A profoundly affects both the physical and emotional well-being of affected children and their families (3). Infants commonly experience irritability, sleep disturbances, chronic pain, prolonged crying episodes, and recurrent vomiting (18). The intensive care required for these children significantly affects caregivers’ QoL, leading to increased stress, anxiety, and depression (3). A recent survey explored the impact of Olipudase-alfa on disease burden from the caregivers’ perspective (22). Participants recognized the positive effects of Olipudase-alfa but expressed concerns about its inability to address the neurological aspects of the disease, continuing to worry about their child's progressive decline. In our case, caregivers reported feelings of stress and frustration as they witnessed their child struggling to lead a normal life, experiencing depression due to the life-threatening nature of the condition, and anxiety about the uncertain disease course, constantly fearing worsening morbidity and death. Despite recognizing the progressive neurodegeneration and the therapy's limitations, caregivers of our patient have not considered terminating treatment finding relief in the reduced disease burden observed in their child.

## 4 Conclusions

Olipudase-alfa is indicated for treating non-central nervous system manifestations of ASMD, as it does not cross the blood-brain barrier, as known for other ERTs. Differentiating between chronic and acute neurovisceral phenotypes in early childhood can be challenging. Therefore, deciding whether to start ERT in an ASMD child at an early stage, especially when clear neurocognitive regression is not evident, may be complex. The drug information leaflet from the European Medicines Agency (EMA) and the Italian Medicines Agency (AIFA) indicates Olipudase-alfa approved only for pediatric and adult patients with type A/B or type B ASMD. In contrast, the FDA's prescribing information's not limit treatment based on phenotype. Our experience with an infant exhibiting a neurovisceral phenotype, treated before neurological symptoms developed, demonstrated safety and effectiveness in achieving significant systemic clinical outcomes. This resulted in a positive impact on both the child's and family's overall quality of life, prompting reflection on therapeutic restrictions based solely on precise clinical phenotype definition.

## Data Availability

The raw data supporting the conclusion of this article will be made available by the corresponding author, upon reasonable request.

## References

[1] Schuchman EH Desnick RJ. Types A and B Niemann–Pick disease. Mol Genet Metab. (2017) 120:27–33. 10.1016/j.ymgme.2016.12.008 28164782 PMC5347465

[2] Zampieri S Filocamo M Pianta A Lualdi S Gort L Coll MJ SMPD1 mutation update: database and comprehensive analysis of published and novel variants. Hum Mutat. (2016) 37:139–47. 10.1002/humu.22923 26499107

[3] Geberhiwot T Wasserstein M Wanninayake S Bolton SC Dardis A Lehman A Consensus clinical management guidelines for acid sphingomyelinase deficiency (Niemann–Pick disease types A, B and A/B). Orphanet J Rare Dis. (2023) 18:85. 10.1186/s13023-023-02686-6 37069638 PMC10108815

[4] Wasserstein M Lachmann R Hollak C Arash-Kaps L Barbato A Gallagher RC A randomized, placebo-controlled clinical trial evaluating olipudase alfa enzyme replacement therapy for chronic acid sphingomyelinase deficiency (ASMD) in adults: one-year results. Genet Med. (2022) 24:1425–36. 10.1016/j.gim.2022.03.021 35471153

[5] Diaz GA Giugliani R Guffon N Jones SA Mengel E Scarpa M Long-term safety and clinical outcomes of olipudase alfa enzyme replacement therapy in pediatric patients with acid sphingomyelinase deficiency: two-year results. Orphanet J Rare Dis. (2022) 17(17):437. 10.1186/s13023-022-02587-0 36517856 PMC9749157

[6] Waelti S Fischer T Wildermuth S Leschka S Dietrich T Guesewell S Normal sonographic liver and spleen dimensions in a central European pediatric population. BMC Pediatr. (2021) 21(21):276. 10.1186/s12887-021-02756-3 34116649 PMC8194166

[7] Deodato F Boenzi S Taurisano R Semeraro M Sacchetti E Carrozzo R The impact of biomarkers analysis in the diagnosis of Niemann–Pick C disease and acid sphingomyelinase deficiency. Clin Chim Acta. (2018) 486:387–94. 10.1016/j.cca.2018.08.039 30153451

[8] Pan YW Tsai MC Yang CY Yu WH Wang B Yang YJ Enzyme replacement therapy for children with acid sphingomyelinase deficiency in the real world: a single center experience in Taiwan. Mol Genet Metab Rep. (2023) 18:34. 10.1016/j.ymgmr.2023.100957 36873248 PMC9979262

[9] Teixeira LF Dornelles AD Poswar F Schwartz IVD. Efficacy and safety of olipudase alfa in children: a real-life case report. Mol Genet Metab. (2024) 141(2):107824. 10.1016/j.ymgme.2023.107824

[10] McGovern MM Wasserstein MP Aron A Desnick RJ Schuchman EH Brodie SE. Ocular manifestations of Niemann–Pick disease type B. Ophthalmology. (2004) 111:1424–7. 10.1016/j.ophtha.2003.10.034 15234149

[11] Vélez Pinos PJ Saavedra Palacios MS Colina Arteaga PA Arevalo Cordova TD. Niemann-Pick disease: a case report and literature review. Cureus. (2023) 15(1):e33534. 10.7759/cureus.33534 36779112 PMC9906968

[12] Simonaro CM Desnick RJ McGovern MM Wasserstein MP Schuchman EH. The demographics and distribution of type B niemann-pick disease: novel mutations lead to new genotype/phenotype correlations. AJHG. (2002) 71:1413–9. 10.1086/345074 12369017 PMC378582

[13] Irun P Mallén M Dominguez C Rodriguez-Sureda V Alvarez-Sala LA Arslan N Identification of seven novel SMPD1 mutations causing Niemann–Pick disease types A and B. Clin Genet. (2013) 84:356–61. 10.1111/cge.12076 23252888

[14] Ricci V Stroppiano M Corsolini F Di Rocco M Parenti G Regis S Screening of 25 Italian patients with Niemann–Pick A reveals fourteen new mutations, one common and thirteen private, in SMPD1. Hum Mutat. (2004) 24:105. 10.1002/humu.9258 15221801

[15] Brown AJ Jessup W. Oxysterols: sources, cellular storage and metabolism, and new insights into their roles in cholesterol homeostasis. Mol Aspects Med. (2009) 30(3):111–22. 10.1016/j.mam.2009.02.005 19248801

[16] McGovern MM Aron A Brodie SE Desnick RJ Wasserstein MP. Natural history of type A Niemann–Pick disease: possible endpoints for therapeutic trials. Neurology. (2006) 66(66):228–32. 10.1212/01.wnl.0000194208.08904.0c 16434659

[17] Wang NL Lin J Chen L Lu Y Xie XB Abuduxikuer K Neonatal cholestasis is an early liver manifestation of children with acid sphingomyelinase deficiency. BMC Gastroenterol. (2022) 22(1):227. 10.1186/s12876-022-02310-0 35534800 PMC9088046

[18] McGovern MM Dionisi-Vici C Giugliani R Hwu P Lidove O Lukacs Z Consensus recommendation for a diagnostic guideline for acid sphingomyelinase deficiency. Genet Med. (2017) 19(9):967–74. 10.1038/gim.2017.7 28406489 PMC5589980

[19] Kaplan P Baris H De Meirleir L Di Rocco M El-Beshlawy A Huemer M Revised recommendations for the management of gaucher disease in children. Eur J Pediatr. (2013) 172(4):447–58. 10.1007/s00431-012-1771-z 22772880

[20] Goker-Alpan O Schiffmann R Park JK Stubblefield BK Tayebi N Sidransky E. Phenotypic continuum in neuronopathic gaucher disease: an intermediate phenotype between type 2 and type 3. J Pediatr. (2003) 143(2):273–6. 10.1067/S0022-3476(03)00302-0 12970647

[21] Weiss K Gonzalez A Lopez G Pedoeim L Groden C Sidransky E. The clinical management of type 2 gaucher disease. Mol Genet Metab. (2015) 114(2):110–22. 10.1016/j.ymgme.2014.11.008 25435509 PMC4312716

[22] Raebel EM Wiseman S Donnelly C Mathieson T Pountney J Crowe J Real-life impacts of olipudase alfa: the experience of patients and families taking an enzyme replacement therapy for acid sphingomyelinase deficiency. Orphanet J Rare Dis. (2024) 19(1):36. 10.1186/s13023-024-03020-4 38303068 PMC10835881

